# Neuron-Derived Extracellular Vesicles Modulate Microglia Activation and Function

**DOI:** 10.3390/biology10100948

**Published:** 2021-09-22

**Authors:** Hui Peng, Brock T. Harvey, Christopher I. Richards, Kimberly Nixon

**Affiliations:** 1 Department of Pharmaceutical Sciences, College of Pharmacy, University of Kentucky, 789 South Limestone Street, Lexington, KY 40536, USA; 2 Department of Chemistry, University of Kentucky, 212 Chemistry-Physics Building, Lexington, KY 40536, USA; Brock_harvey@uky.edu (B.T.H.); chris.richards@uky.edu (C.I.R.); 3 Division of Pharmacology & Toxicology, College of Pharmacy, The University of Texas at Austin, Austin, TX 78712, USA; Kim.nixon@austin.utexas.edu

**Keywords:** extracellular vesicle, neuron, microglia activation, inflammation, cytokine

## Abstract

**Simple Summary:**

In this study we investigated how neuron-derived extracellular vesicles (NDEVs) mediate neuroimmune regulation in primary cell culture systems. Rat cortical neurons released EVs that improved microglial survival and inhibited the expression of activation markers on microglia. Furthermore, NDEVs reduced the LPS-induced proinflammatory response and promoted an anti-inflammatory response. Thus, neurons critically regulate microglia activity and control inflammation via EV-mediated neuron–glia communication.

**Abstract:**

Microglia act as the immune cells of the central nervous system (CNS). They play an important role in maintaining brain homeostasis but also in mediating neuroimmune responses to insult. The interactions between neurons and microglia represent a key process for neuroimmune regulation and subsequent effects on CNS integrity. However, the molecular mechanisms of neuron-glia communication in regulating microglia function are not fully understood. One recently described means of this intercellular communication is via nano-sized extracellular vesicles (EVs) that transfer a large diversity of molecules between neurons and microglia, such as proteins, lipids, and nucleic acids. To determine the effects of neuron-derived EVs (NDEVs) on microglia, NDEVs were isolated from the culture supernatant of rat cortical neurons. When NDEVs were added to primary cultured rat microglia, we found significantly improved microglia viability via inhibition of apoptosis. Additionally, application of NDEVs to cultured microglia also inhibited the expression of activation surface markers on microglia. Furthermore, NDEVs reduced the LPS-induced proinflammatory response in microglia according to reduced gene expression of proinflammatory cytokines (TNF-α, IL-6, MCP-1) and iNOS, but increased expression of the anti-inflammatory cytokine, IL-10. These findings support that neurons critically regulate microglia activity and control inflammation via EV-mediated neuron–glia communication. (Supported by R21AA025563 and R01AA025591).

## 1. Introduction

Microglia, one of three types of glial cells found in the CNS, though of myeloid origin [1], act as the brain’s primary immune cells. As such, microglia play varying roles in development versus damage, infection, aging, or neurodegenerative diseases [2]. Microglia display a variety of functional states in the healthy brain but especially with neuropathology. For example, under resting conditions, microglia exhibit a ramified morphology allowing for active surveillance of their environment [3,4]. Upon homeostatic disturbances, microglia adopt reactive profiles, which range across a spectrum from classical activation (proinflammatory, M1-like) to alternative activation (anti-inflammatory, M2-like) phenotypes. Proinflammatory activated “M1-like” microglia produce cytokines, chemokines and radical species, which contribute to brain inflammation and further brain damage [5,6]. “M2-like” microglia, however, produce anti-inflammatory cytokines and growth factors that are associated with reparative processes or resolution of damage [6]. Both the quiescent and activated states of microglia are controlled by cell-autonomous mechanisms, such as epigenetic, microRNA and other extracellular signals [2]. Growing evidence supports that neurons play a crucial role controlling microglia function. Neurons constitutively express or secrete signaling molecules such as CD200, CD47, and CX3CL1 that bind corresponding receptors found on microglia (CD200R, C172a, and CX3CR1) to keep microglia in their quiescent state and/or inhibit their proinflammatory functions [7,8,9]. In addition to providing inhibitory signals through the secretion of soluble factors or cell-to-cell contact, neurons also release extracellular vesicles (EVs) that play an important role in neuron–glia communication [10,11,12].

EVs have recently emerged as a means of cell-to-cell communication in the brain [13,14,15]. EVs are heterogeneous, cell-derived membranous vesicles, that are classified based on their size and means of biogenesis [13,14]. EVs include endosome-derived exosomes (50–200 nm diameter), irregularly shaped, larger microvesicles (MVs; 100–1000 nm diameter), and apoptotic bodies (diameters up to 5 um). EVs carry a broad spectrum of proteins, lipids, and a range of nucleic acids including messenger RNA (mRNA), microRNA (miRNA), and non-coding RNA [16]. EVs play active roles in development and normal brain function but also in disease states via horizontal transfer of genetic information, proteins, and lipids between cells without direct cell-to-cell contact [13,14,15]. In the CNS, EVs can be released from all cell types including microglia, oligodendrocytes, astrocytes, and neurons, and have been proposed to contribute to the neuron–glia communication in various physiological processes of the nervous system [17,18,19]. In particular, exosomes are released by cultured neurons [11,20] and can be internalized by microglia. Neuronal exosomes suppress the proinflammatory activation of microglia (M1) via their cargo, especially microRNAs, present in exosomes derived from spinal cord neurons [12]. However, the effects of NDEVs on microglia activation and function are not fully understood.

In this study, we hypothesize that NDEVs function as intercellular communicators between neurons and microglia and provide inhibitory signals under both normal and immune-activated conditions. Our in vitro studies demonstrate the role of NDEVs in microglia survival and activation which suggests that NDEVs may regulate microglia activation and control inflammation. These findings have implications for neurodegenerative and psychiatric disorders where microglia likely play a role.

## 2. Materials and Methods

### 2.1. Cells

All procedures were in accordance with the Guide for the Care and Use of Laboratory Animals and were approved by the University of Kentucky Institutional Animal Care and Use Committee prior to the start of experiments. Primary cortical neurons were prepared and cultured from cortex of embryonic day 18–19 rat embryos as described previously with modification [21]. Briefly, cortices were dissected out and incubated with 0.25% trypsin in Hank’s Balanced Salt Solution (Thermo Fisher, Waltham, MA, USA) for 15 min at 37 °C, followed by mechanical dissociation. Single-cell suspensions were plated onto T75 flasks coated with poly-D-lysine (50 μg/mL, Millipore Sigma, St. Louis, MO, USA) at a density of 5 × 10^5^ cells/cm^2^ in Neurobasal medium containing B27 supplement (1×, Thermo Fisher) and penicillin-streptomycin (1×, Thermo Fisher). Cells were incubated at 37 °C and 5% CO_2_ in a humidified incubator.

Microglia cultures were prepared as described previously [22]. Briefly, cortices were obtained from postnatal day 2–3 rat pups, stripped of meninges, dissociated with a pipette, and passed through a 100 μm cell strainer. The cell suspension was seeded into T75 tissue culture flasks (one rat pup brain per flask) and maintained in Dulbecco’s Modified Eagle’s Medium (Thermo Fisher, Waltham, MA, USA) supplemented with 10% Fetal Bovine Serum (Thermo Fisher) and 1% penicillin/streptomycin and grown as a mixed glia culture for 7–10 days. After mixed glia cultures were completely confluent, flasks were sealed with parafilm and shaken gently at 100 rpm for 1 h at 37 °C to detach microglia. Next, microglia in suspension were removed from mixed culture and pelleted at 400× *g* for 5 min at 4 °C. The purity of microglia was determined by immunocytochemical staining (Supplementary Figure S1). Results showed that over 99.9% of cells were Iba-1+ (microglia-specific marker), with less than 0.1% of cells Glial Fibrillary Acidic Protein (GFAP+; astrocyte-specific marker), and no NG2+ or MBP+ (oligodendrocyte-specific markers) cells were observed in the culture. Cells were then plated at a density of 2 × 10^5^ cells per mL for further treatment.

### 2.2. Extracellular Vesicle Purification

Neuron-derived extracellular vesicles (NDEVs) were prepared from neuron-conditioned medium by differential ultracentrifugation as described previously [23]. Briefly, neuron-conditioned medium was collected from neuronal cultures that were maintained for 5–7 days in vitro and subjected to serial differential centrifugations at 300× *g* for 10 min and 2000 × g for 20 min at 4°C to remove dead cells and cell debris. Supernatants were then centrifuged at 10,000× *g* (Beckman XL 90 ultracentrifuge; 70 TI Rotor; k-factor, 44) for 30 min at 4°C to pellet large EVs (L-EVs) [23]. The L-EV pellet was washed with phosphate buffered saline (PBS) and subjected to an additional centrifugation at 10,000× *g* for 30 min at 4°C. Large EV pellets were then resuspended in PBS and stored at −80 °C in aliquots. Small EVs (S-EVs) remaining in the medium were then pelleted by ultracentrifugation at 100,000× *g* (Beckman XL 90 ultracentrifuge; 70 TI Rotor; k-factor, 44) for 70 min at 4°C. The S-EV pellet was washed with PBS and then subjected to another centrifugation at 100,000× *g* (Beckman XL 90 ultracentrifuge; 70 TI Rotor; k-factor, 44) for 70 min at 4°C. S-EVs were resuspended in PBS and stored at −80°C in aliquots or proteins were extracted with RIPA buffer (Thermo Fisher) for further analysis by Western blotting.

EV concentration and size distribution were measured using multiple particle tracking with a Nanosight NS300 (Malvern Panalytical, Malvern, UK) equipped with a 488 nm laser. Multiple tracking analysis measures the diffusion time of individual nanoparticles to determine the size and concentration. All samples were measured at least 5 times for a duration of 60 s each with a minimum of 200 valid tracks per recording. Analysis was performed using Nanosight 3.4 software. Instrument calibration was verified using 100 nm polystyrene standard beads. EV protein concentration was measured via a bicinchoninic acid (BCA) protein assay kit (Pierce, Rockford, IL) following the manufacturer’s instructions: (S-EVs: 0.800 ± 0.322 × 10^9^/mg; L-EVs: 0.291 ± 0.047 × 10^9^/mg).

For in vitro microglia treatment experiments, EVs (0–10 μg/mL) were suspended in microglia culture medium and added into microglia culture (1.5 to 2 × 10^5^ cells per well) for 24 h (based on pilot studies), followed by incubation with or without Lipopolysaccharide (Millipore Sigma) for 8 h for RNA or 24 h for protein. Microglia were collected 24–32 h following EV treatment for flow cytometric analysis or RNA extraction. Microglia supernatant was collected 48 h following EV treatment for cytokine ELISA.

### 2.3. Western Blot

Western blots were performed on EV protein or whole neuronal cell lysate extracted using RIPA buffer containing a protease inhibitor cocktail (Thermo Scientific, Rockford, IL). Quantification of the isolated protein was achieved using a BCA protein assay according to the manufacturer’s instructions. A total of 10 μg of protein was boiled in 4× Laemmli Sample Buffer (Invitrogen) supplemented with 2% β-mercaptoethanol for 5 min before being loaded for electrophoresis on 10% polyacrylamide gels. The resolved proteins were then transferred onto nitrocellulose membranes (BioRad, Hercules, CA, USA) with Precision Plus Protein Dual Color Standards (Bio-Rad, #1610374) on the side well. Membranes were blocked in 5% skim milk powder in Tris-buffered saline (TBS), and blotted with primary antibodies (Table 1) overnight at 4°C on a shaker. Membranes were then incubated with goat anti-rabbit or goat anti-mouse Ig G IR800 secondary antibody (1:20,000 dilution; Azure Biosystems, Inc., Dublin, CA, USA) for 1 h at room temperature and then visualized using Sapphire Biomolecular Imager (Azure Biosystems). Blots were quantified by ImageJ software (NIH, Bethesda, MD, USA).

### 2.4. MTT Assay

The MTT [3-(4,5-dimethylthiazol-2-yl)-2,5-diphenyltetrazolium bromide] assay is used to measure cellular metabolic activity as an indicator of cell viability. Microglia were treated with different concentrations (0, 1, 5, 10 μg/mL) of S-EVs or L-EVs for 24 h. MTT was added to make up a final concentration of 0.5 mg/mL in medium and cells were incubated for 1 h at 37°C in 5% CO_2_. Cells were dissolved in dimethyl sulphoxide (DMSO) and absorbance at 490/570 nm was determined in a plate reader (M5, Molecular Devices, Sunnyvale, CA, USA).

### 2.5. LDH Assay

Lactate dehydrogenase (LDH) is a cytosolic enzyme released into the cell culture media upon damage to the plasma membrane. Microglia were incubated with S-EVs for 24 h then LDH release into the medium was measured by a Pierce LDH cytotoxicity Assay Kit (Thermo Scientific) following exactly the manufacturer’s instructions. Absorbance at 562 nm was measured in an M5 plate reader.

### 2.6. Propidium Iodide Flow Cytometric Assay

Propidium iodide (PI) flow cytometric assays are well-accepted methods for the evaluation of cell cycle and apoptosis [24]. Microglia were detached from culture wells by trypsin and fixed in 66% ethanol on ice. Cells were then incubated in PI (50 μg/mL) + RNase (10 μg/mL) at 37°C in the dark for 30 min. Samples were run on an Attune Acoustic Focusing Cytometer (ABI, Carlsbad, CA) and PI fluorescence was collected in FL2 channel. DNA content was quantified in a histogram plot to delineate cells in G1 (2N), DNA synthesis (2N-4N), mitotic (4N), and apoptotic stages (<2N).

### 2.7. Microglia Staining and Flow Cytometry

Microglia were scraped from culture wells and suspended in incubation buffer (50 μL; 1 × PBS + 0.1%BSA) for 30 min on ice. Cells were incubated with anti-CD32 (BD Bioscience, San Jose, CA) to block F_c_ receptors on microglia for all assays except when used to assess CD32 immunoreactivity. Cells were then stained with fluorescent conjugated antibodies on ice for 30 min in order to assess microglia purity (mouse anti-rat CD11b-FITC, #554982, BD Bioscience, San Jose, CA; mouse anti-rat-CD45-APC, #17-0461-82, eBioscience, San Diego, CA, USA) and state of M1 activation (mouse anti-rat: MHC-II-PE #554929 and CD32-PE, #552189, BD Bioscience). For alternative/M2-like activation, cells were incubated in rabbit anti-rat CD206 (#ab64693, Abcam, Cambridge, MA) for 30 min followed by incubation with donkey anti-rabbit-PE secondary antibody (#12-4739-81, BD Bioscience) for 30 min. Cells were washed with PBS, fixed with IC fixation buffer (Invitrogen by ThermoFisher) and analyzed on an Attune Acoustic Focusing Cytometer (ThermoFisher). Prior to each run, the flow cytometer was calibrated with commercially available beads (ThermoFisher). Fluorescence spillover compensation values were then generated from both non-stained cell populations as well as single-color staining controls. Isotype controls were also utilized to exclude any the non-specific binding of the antibodies. For each sample, 1 × 10^4^ events were collected.

### 2.8. RNA Isolation and Real-Time PCR

After EVs and/or LPS treatment, microglia were lysed with TRIZOL Reagent (Life Technologies, Carlsbad, CA, USA) and total RNA was extracted using a mirVana miRNA Isolation Kit (ThermoFisher, Waltham, MA, USA) according to the manufacturer’s instructions. Real-time RT-PCR was performed with Assays-on-Demand primers [TNF-α (Rn00562055_m1), IL-6 (Rn01410330_m1), MCP-1 (Rn00580555_m1), iNOS (Rn00561646_m1), IL-10 (Rn01483988_g1), Applied Biosystems Inc.], using a one-step quantitative Real-time RT-PCR system (ThermoFisher, Waltham, MA, USA). The housekeeping gene, glyceraldehyde-3-phosphate dehydrogenase (GAPDH, Rn01462661_g1) was used as an internal control. Data were analyzed by calculating the differences between the delta cycle values for the EV/LPS treatments and control conditions (double delta cycle analysis) as previously described [25]. Results were expressed as fold difference as compared to no EV treatment control.

### 2.9. Enzyme-Linked Immunosorbent Assay (ELISA)

ELISA for TNF-α and IL-6 were performed according to the manufacturer’s instructions (DuoSet ELISA for TNF-α and IL-6, R&D Systems, Minneapolis, MN, USA). Briefly, 96-well plates were coated with capture antibodies for TNF-a or IL-6 in PBS overnight at room temperature (RT). Plates were blocked with 1% bovine serum albumin (BSA) in PBS for 2 h at RT, following which samples or standards were added and incubated for 2 h at RT or overnight at 4 °C. Adhering antigen was detected by incubation with biotin-conjugated detection antibody for 2 h at RT followed by horseradish peroxidase-conjugated streptavidin for 20 min. Then, 100 μL of Substrate Solution (1:1 mix of H_2_O_2_ and Tetramethylbenzidine) were added to each well, followed by 50 μL of Stop Solution (2N H_2_SO_4_). Optical density was determined using a microplate reader (BioTec, Winoosk, VT, USA) set to 450 nm and wavelength correction set to 540 nm.

### 2.10. Statistical Analysis

All of the resulting raw data were compiled in Excel and then graphed and analyzed in Prism (v7, GraphPad Software, San Diego, CA, USA). Unless stated otherwise, all values are reported as mean ± S.D. with n indicating the number of replicates. Flow cytometry data were compared via Student’s t-test (for two groups) or one-way ANOVA with Holm–Sidak’s posthoc test (all groups versus control) or Tukey’s (3 groups, all pairwise comparisons) posthoc test. Data for real time RT-PCR were compared using two-way ANOVA for EV treatment and LPS as factors with Tukey’s posthoc tests. Statistical significance was accepted at a *p* < 0.05.

## 3. Results

### 3.1. EVs Derived from Neurons Improve Microglia Survival

**Figure 1:**
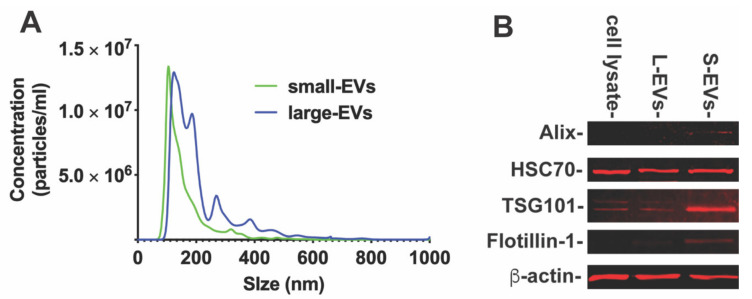
(**A**). Particle size distribution of small- and large-EVs isolated from cultured primary neurons by Nanopartical tracking analysis showing the size versus concentration of particles. (**B**). The Western blot analysis of characteristic markers of EVs. The characteristic markers (Alix, TSG101 and Flotillin) were more highly expressed in S-EVs than L-EVs and neuronal cell lysate. Band intensity was analyzed by ImageJ software as shown in Table 2.

To determine if NDEVs contribute to neuron–microglia intercellular communication, we obtained EVs through serial steps of ultracentrifugations from rat cortical neuronal cultures as described in Section 2. The size distribution of small or large EVs (S-EVs or L-EVs) was analyzed by nanoparticle tracking analysis (NTA, Figure 1A). NTA revealed that S-EVs secreted from neurons were 158.3 ± 75.9 nm in size, with a peak diameter of about 106 nm (Figure 1A). The L-EV isolation was more heterogenous, with multiple peak diameters from 124 to 768 nm (Figure 1A). We then examined the characteristic markers, including Alix, flotillin, TSG101, and HSC70 by Western blotting (Figure 1B). The characteristic markers Alix, flotillin and TSG 101 were highly expressed in S-EVs, while HSC70 was expressed in both S-EVs and L-EVs. These results confirmed that the particles we extracted were EVs.

**Figure 2:**
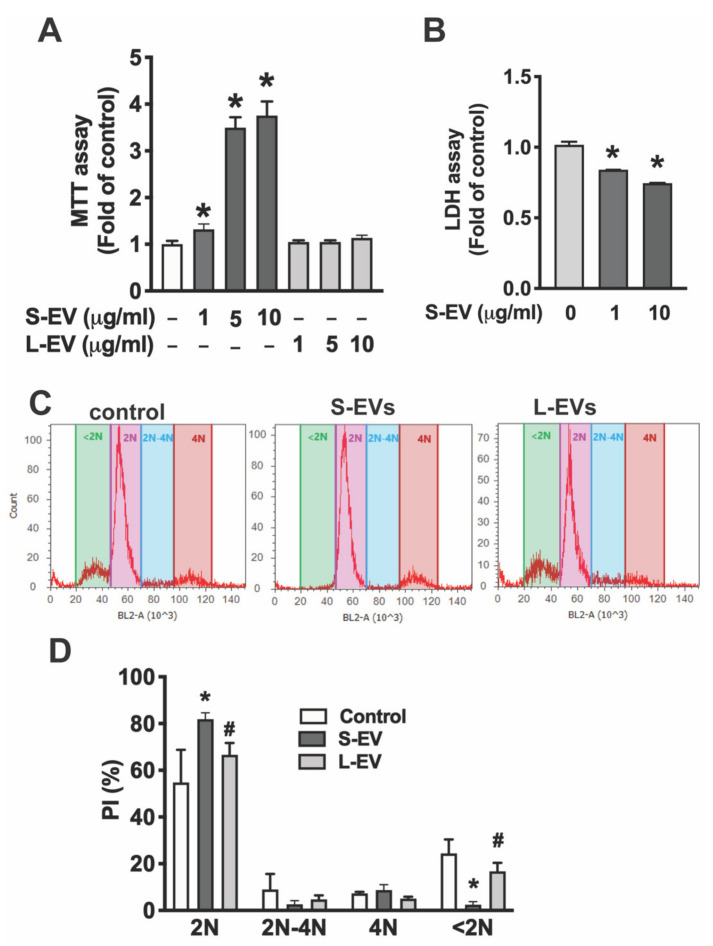
(**A**) Primary microglia were treated with NDEVs purified from rat neuronal cultures for 24 h and cell viability was detected by MTT assay. (**B**) Cell supernatant was collected for LDH assay. Data in A and B are shown as fold of control. (**C**) Flow cytometric analysis of DNA content by PI staining. (**D**) Data are shown as % of cells in G1 stage (2N), DNA synthesis (2N-4N) and mitotic (4N) or apoptotic (<2N). Data are mean values ± SD of three independent experiments.; * *p* < 0.05 versus control via post test. # *p* < 0.05 versus S-EV via post test.

To determine the effect of NDEVs on microglia, primary microglia were treated with of different concentrations of S-EVs (0–10 μg/mL) for 24 h. S-EVs increased microglia cell viability in a dose-dependent manner as indicated by MTT assay [F(6,23) = 283.2; *p* < 0.0001] by 1.32 ± 0.12 fold for 1 μg/mL (*p* < 0.05), 3.5 ± 0.22 fold for 5 μg/mL (*p* < 0.0001), and 3.75 ± 0.30 fold for 10 μg/mL (*p* < 0.0001; Figure 2A). Importantly, this effect is S-EV specific as L-EVs isolated from neuronal culture do not increase microglial cell viability (Figure 2A). To determine if the S-EV-mediated increase of microglial cell viability is through an increase in cell survival, we determined extracellular LDH in the supernatant of microglia treated with or without neuronal S-EVs. Results showed that S-EVs reduced LDH release from microglia [F(2,7) = 548.7; *p* < 0.0001] with a 16% reduction for 1 μg/mL, (Holm–Sidak: *p* < 0.0001) and 25% reduction for 10 μg/mL (*p* < 0.0001; Figure 2B). To further confirm that the effect of neuronal S-EVs on microglia is through an increase in cell survival but not through inducing cell proliferation, we determined cell cycle status by measuring DNA content using Propidium iodide (PI) staining combined with flow cytometric analysis. PI flow cytometry has also been used widely for the evaluation of cell apoptosis [24]. PI flow cytometry showed neither S-EVs nor L-EVs change cell populations undergoing DNA synthesis (2N-4N), which suggests that they do not promote microglial cell proliferation. In addition, S-EV treatment reduced the apoptotic cell population, <2N, from 24.4 ± 6.0% to 2.9 ± 1.4% [F(2,7) = 32.37; *p* = 0.0003; Tukey’s *p* = 0.0004], while L-EVs had little effect on the apoptotic cell population (22.2 ± 3.0%, Figure 2C,D). These results suggest that S-EVs protect microglial cells from apoptosis but do not increase microglial proliferation.

### 3.2. EVs Derived from Neurons Impact the Activity of Microglia

**Figure 3:**
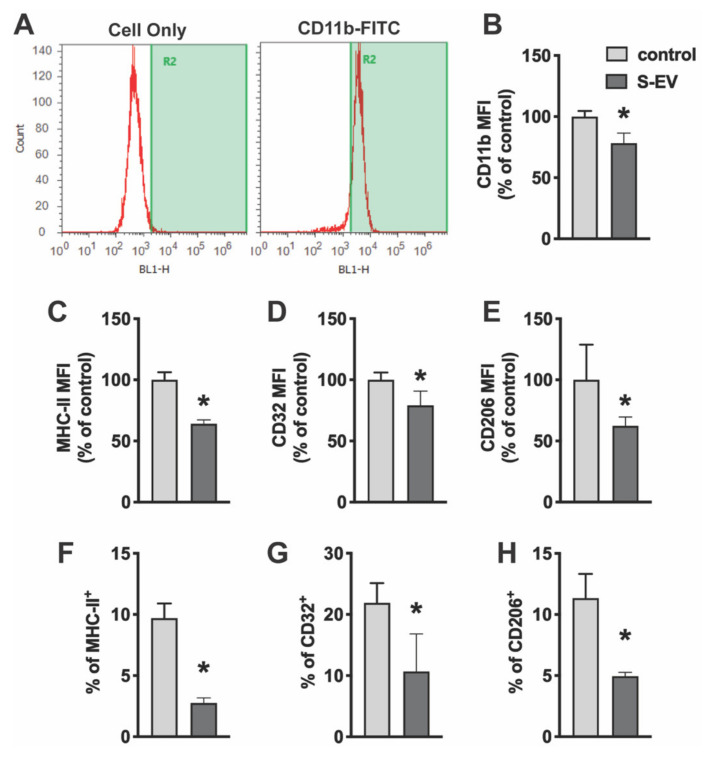
Primary microglia were treated with NDEVs purified from rat neuronal cultures. The expression of microglia/macrophage phenotypic markers, CD11b, CD32, MHC-II and CD206, were detected using an Attune Acoustic Focusing Cytometer. (**A**) Isolated microglia express CD11b. (**B**–**H**) Data presented show mean fluorescent intensity (MFI) of CD11b (**B**), MHC-II (**C**), CD32 (**D**), and CD206 (**E**) expression or percentage of MHC-II (**F**), CD32 (**G**), and CD206 (**H**) expression on microglia with or without S-EV treatment. Data are mean values ± SD of three to six independent experiments. * *p* < 0.05 versus control.

To determine the effect of neuronal EVs on microglia activity, primary microglia cultures were treated with NDEVs purified from rat cortical neuron culture. Twenty-four hours later, microglia were scraped from culture dishes and stained with microglia surface antigens. Fixed cells were then analyzed by flow cytometry. Cells were stained for CD11b (a component of complement receptor 3) to confirm cell purity (Figure 3A). Consistent with immunocytochemical staining (Supplementary Figure S1), these isolated cells were highly enriched for microglia. CD11b is expressed constitutively by microglia and increases to a greater extent upon microglia activation. Results showed that S-EVs reduced mean fluorescence intensity (MFI) of CD11b by 21.8 ± 8.3% (Figure 3B). Microglia activation states can be classified as either M1-like or M2-like based on changes in morphology and/or expression of phenotypic, cell surface antigens [26,27,28]. For example, phenotypic markers such as major histocompatibility complex (MHC) II and CD32 have been used to identify M1-like cells [29]. M2-like microglia, on the other hand, express CD206 (macrophage mannose receptor 1) on the cell membrane. Results showed that S-EV treatment reduced M1-like microglia as indicated by a decrease in the expression frequency of MHC-II^+^ cells (9.7 ± 1.2% in controls vs. 2.8 ± 0.4% in S-EV treated cells, *p* < 0.001; Figure 3F) and CD32^+^ cells (21.9 ± 3.2% in controls vs. 10.7 ± 6.2% in S-EV treated cells, *p* = 0.018; Figure 3G). S-EV treatment also decreased the MFI of MHC-II by 35.9 ± 3.2% (Figure 3C) and CD32 by 20.8 ± 11.7%. L-EVs, however, had little effect on the MFI of CD11b or CD32, or the expression frequency of CD32 (Supplemental Figure S2). We also observed a decrease of M2-like microglia as indicated by decrease of CD206^+^ cells: 11.3 ± 2.0% in controls vs. 5.0 ± 0.3% in S-EV treated cells (*p* < 0.001; Figure 3H), as well as a 37.6 ± 7.3% reduction of CD206 MFI (Figure 3E).

### 3.3. Neuronal EVs Suppress LPS-Induced Microglia Activation

**Figure 4:**
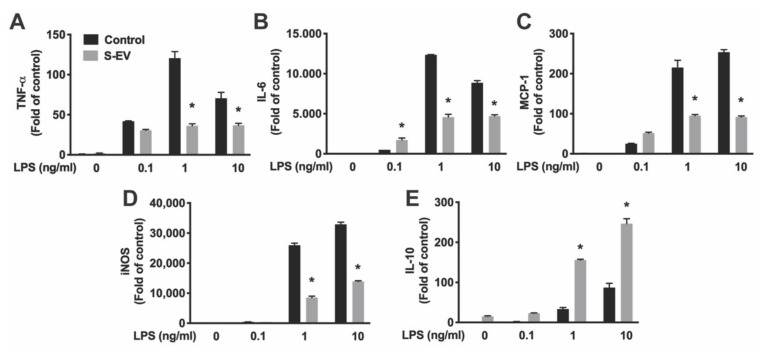
Total RNA was extracted from EV-treated microglia. mRNA expression of TNF-α (**A**), IL-6 (**B**), MCP-1 (**C**), iNOS (**D**) and IL-10 (**E**) was determined by real-time RT-PCR and presented as folds of control. PCR was run in triplicate and data were presented as mean fold of control ± SD. Data represented three independent experiments and was analyzed by ANOVA. Statistics are listed in Table 3. * *p* < 0.05 versus respective control with LPS.

**Figure 5:**
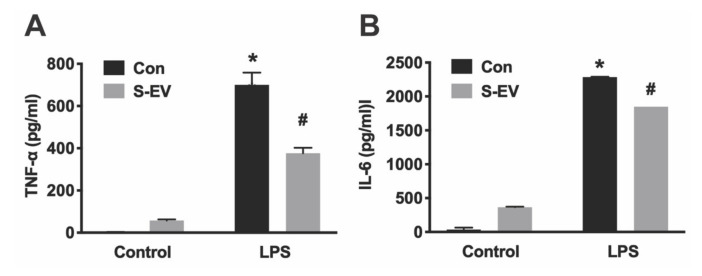
Primary microglia were pre-incubated with S-EVs for 24 h and then treated with LPS for 24 h. Culture supernatant was collected to determine cytokine [TNF-α (**A**) and IL-6 (**B**)] expression by ELISA. Data represented three independent experiments and was analyzed by ANOVA. * *p* < 0.05 versus control. # *p* < 0.05 versus non S-EV.

Lipopolysaccharide (LPS, the major component of the outer membrane of Gram-negative bacteria) activates microglia/macrophages and induces proinflammatory activation, which produces proinflammatory cytokines and inducible nitric oxide synthase (iNOS) [30]. Primary microglia were pre-incubated with S-EVs for 24 h and then treated with LPS (0.1–10 ng/mL) for 8 h. Total RNA were extracted, and the levels of mRNA encoding pro-inflammatory cytokine/chemokines (IL-6, TNF-α, and MCP-1), iNOS, and anti-inflammatory cytokine (IL-10) were quantified by real-time RT-PCR. Results showed that LPS induced concentration-dependent increases of TNF-α, IL-6, MCP-1, IL-10, and iNOS expression indicated by main effects of LPS concentrations (Table 3). S-EV pre-treatment inhibited LPS-induced proinflammatory cytokines TNF-α and IL-6 (Figure 4A,B), chemokine MCP-1 (Figure 4C), and iNOS expression (Figure 4D), but promoted anti-inflammatory cytokine, IL-10, expression in microglia as indicated by main effects of EV treatment and a significant interaction of LPS concentration and EV treatment (Table 3; Figure 4E). To determine if L-EVs similarly suppressed LPS-induced microglia activation as S-EVs, primary microglia were pre-incubated with S-EVs or L-EVs for 24 h and then treated with LPS (1 ng/mL) for 8 h. L-EV pre-treatment did not inhibit LPS-induced TNF-α, MCP-1, or iNOS expression, and did not promote IL-10 expression in microglia (Supplementary Figure S3). In addition, primary microglia were pre-incubated with S-EVs for 24 h and then treated with LPS for 24 h. Cytokines (TNF-α and IL-6) released into culture supernatant were determined by ELISA (Figure 5). Results showed that S-EV pre-treatment inhibited LPS-induced proinflammatory cytokine TNF-α and IL-6 expression. These data suggest that neuronal EVs modulate innate immunity in the brain, dampening pathogenic M1 microglia, and point to a possible mediator for suppression of neuroinflammation.

## 4. Discussion

The interactions between neurons and microglia represent a key process of neuroimmune regulation with potential implications for the regulation of CNS integrity in neurodegenerative and psychiatric disease [31,32,33,34]. Secretion of exosomes from cultured primary neurons has been observed previously [11,20]. In this study we demonstrated the potential role of neuron derived EVs as a means of intercellular signaling in neuron–microglia communication. We isolated EVs from rat cortical neuronal culture and exposed microglia to these NDEVs. Here, we show that supernatants of these primary cortical culture contained small EVs of a composition and size typical of exosomes. S-EVs promoted microglia survival and inhibited microglia activation marker expression, both effects of which were S-EV specific as large EVs did not have the same effects on microglia. We also found that incubating microglia with S-EVs inhibited LPS-induced pro-inflammatory cytokine expression. These results indicate that S-EVs released by neurons regulate microglia reactivity and control LPS-induced proinflammatory microglia activation. Considering the importance of microglia reactivity in both physiological and pathological conditions, these results suggest a new pathway of microglia regulation.

Our understanding of the role of exosomes as an important mechanism for intercellular communication in the CNS is just beginning to emerge [13,17,18,35]. Exosomes facilitate the transfer of information between cells through their release and shuttling of a cargo of various signaling proteins and coding and/or regulatory RNAs, that are then taken up by target cells. Exosomes, therefore, not only play critical roles in physiological processes, such as synaptic function, nerve regeneration, and neuronal development, but are also implicated in the pathogenesis of a variety of neurodegenerative disorders. For example, exosomes secreted from a variety of cell types have been shown to contain prions or beta-amyloid peptides, which suggests their role in the transmission of toxic proteins in neurodegenerative conditions [36,37]. In addition, they may also contribute to the neuroimmune activities through the shuttling of signaling molecules between neurons and glia [17,18,35].

Neuron–glia communication has been shown to play a critical role in the nervous system in both normal physiological as well as pathological conditions. There is increasing evidence to indicate that neurons are not merely victims of (over)activated microglia but rather control microglial function and activity [2,7]. For example, neurons constitutively express “Off” signals which are thought to keep microglia in a quiescent state. This process aids in maintaining tissue homeostasis, but also restricts pro-inflammatory microglia activity to prevent further damage to the brain [7]. Most of these effects are through the expression of signaling molecules on plasma membranes (CD200, CD47, etc.) or the secretion of soluble ligands (CX3CL1) [2]. Our work further demonstrated that neurons release EVs that may have significant roles in maintaining a homeostatic phenotype of microglia and regulating their activation beyond these mechanisms. Our results showed that extracellular particles with the characteristics of EVs (size distribution and characteristic marker expression) are involved in neuron-to-microglia communication and may deliver cargo from neurons to microglia as evidenced by the functional change of microglia (improved survival, maintaining microglia quiescence and inhibition of over-activation) after S-EV treatment. Thus, the results of this study demonstrate that constitutively produced NDEVs represent a new means of regulating microglia function.

Indeed, NDEVs have been shown to elicit various physiological responses in target microglia. For example, more microglia survived in vitro if they had received small EVs, which suggests that NDEVs may play a protective role and increase microglia tolerance to stress. The roles of NDEVs in control of microglial activation can be divided into two mechanisms: to stabilize microglia in their quiescent state by inhibition of activation mechanisms (as suggested by reduced activation marker expression) in normal conditions and/or antagonize LPS-induced proinflammatory activity. Microglial activation in the normal, healthy brain is constrained by “Off” signals that are constitutively expressed by neurons in the normal brain microenvironment [2,7]. Without these in vivo inhibitory signals and under the exposure of fetal bovine serum in the culture media, microglia in culture are a mixture of M1 or M2-like and non-activated cell populations as indicated by the M1 marker (MHC-II and CD32) and M2 marker (CD206) expression (Figure 2). Here, we showed that small NDEVs inhibit activation markers expressed in microglia under normal culture conditions, which suggests that EVs may contribute to these “Off” signals in the normal brain microenvironment [2]. Under pathological insults, microglia respond as either neurotoxic or neuroprotective depending on the various signals in the microenvironment [2]. For example, LPS induces a neurotoxic microglia response through the release of proinflammatory cytokines and inducing oxidative stress [30]. To further evaluate if NDEVs regulate microglia activation under LPS-induced proinflammatory activation, microglia were exposed to S-EVs and stimulated with LPS. Our results showed that the gene expression pattern is modified in LPS-activated microglia that received S-EVs: LPS-induced pro-inflammatory cytokines/chemokine (IL-6, TNF-α, and MCP-1) and iNOS gene expression are inhibited, consistent with a recent study in spinal cord [12]. While mRNA expression may not always mirror protein expression, we confirmed that S-EVs inhibit LPS-induced proinflammatory cytokine (TNF-α and IL-6) secretion with ELISA (Figure 5).

We also demonstrated that NDEVs increased a potent, anti-inflammatory cytokine, IL-10, gene expression. IL-10 limits host inflammatory response to pathogens thus preventing inflammation. Although it has been shown that IL-10 inhibits LPS-induced proinflammatory cytokine secretion [38], whether or not IL-10 contributes to NDEV-mediated inhibition of LPS-induced proinflammatory cytokine production will need further investigation. In addition, we observed that S-EVs change microglia phenotypes from activated M1 or M2-like microglia to non-activated states in normal culture conditions, however, it remains unknown whether NDEVs inhibit LPS-induced proinflammatory cytokines and iNOS production through change a microglia phenotype or through the regulation of specific genetic pathways. The regulation of microglia phenotype and cytokine production may occur through different mechanisms. A thorough RNA sequencing analysis of regulated genes will be helpful to extend this work into mechanistic directions. Subsequent studies in cytokine knockout models would then be important for determining the specific genetic pathway effect versus phenotypic output. Thus, NDEVs dampen microglia immune reactivity induced by LPS and prevent the development of excessive and uncontrolled stimulation of microglia that may lead to secondary neuronal damage.

The underlying mechanism behind the NDEV effect on microglia is not fully understood, but their various cargos provide clues to these effects. Neuronal EVs may express the inhibitory signaling molecules on their membrane, and thus keep microglia inactivated. The expression of these signaling molecules in neuronal EVs and their roles in maintaining a homeostatic phenotype of microglia needs further confirmation. In addition to protein cargo, miRNAs may also contribute to the effects of NDEVs on microglia. A recent study demonstrated that neuronal exosomes shuttle microRNA-124-3p to microglia and mediate the suppression of M1 microglia and A1 astrocyte activation after spinal cord injury [12]. Our preliminary miRNA sequencing analysis reveals that miRNAs known to regulate microglia/macrophage function such as miR-125b, miR-9a, miR-let-7a, miR-let-7c, miR-30a, and miR-181c are highly expressed in NDEVs [39] (unpublished observations; Peng et al., in preparation). Whether or not NDEVs shuttle these miRNAs to microglia and mediate the suppression of LPS-mediated microglia activation will need further investigation. The distinct function of S-EVs and L-EVs on microglia may be attributed to their differential cargos. Proteomic analyses have indicated that exosomes are enriched with receptors and kinases that mediate signaling in immune regulation, whereas MVs are more implicated in protein translation [13,40]. Although current EV isolation techniques do not distinguish exosomes versus MVs, a thorough analysis of the cargos (both protein and nuclei acid) of S-NDEVs and L-NDEVs will help to identify the components that contribute to their differential function on microglia. Future work combining next-generation RNA sequencing, proteomics, and bioinformatic analysis is needed to identify the specific RNAs and proteins present in NDEVs that mediate this effect of NDEVs on microglia function.

## 5. Conclusions

In summary, this study investigated a novel regulatory mechanism in neuron-to-microglia communication. These data provide new insight into EV-mediated regulation of microglia function and activation under pro-inflammatory conditions. The specific components in EVs that contributed to these effects are unclear, but neuronal EVs contain numerous signaling molecules, including proteins and RNAs, that play significant roles in neuron-to-microglia communication. Ultimately, these results contribute to our understanding of the mechanisms of neuronal regulation of microglia activation, a phenomenon that has major implications for our understanding of—and the development of new therapies for—neurodegenerative and psychiatric disease.

## Figures and Tables

**Table 1:** Primary antibodies.

Antibody	Host Species	Cat. Number	Dilution	Resource
Anti-TSG 101	Rabbit	ab125011	1:1000	Abcam
Anti-Flotillin-1	Rabbit	#18634	1:1000	Cell Signaling
Anti-Alix	Mouse	#2171	1:1000	Cell Signaling
Anti-HSC70	Mouse	#2171	1:1000	Cell Signaling
Anti-β-Actin	Rabbit	#4970	1:10,000	Cell Signaling
Anti-CD11b-FITC	Mouse	#554982	1:100	BD Bioscience
Anti-CD45-APC	Mouse	#17-0461-82	1:100	BD Bioscience
Anti-MHC-II-PE	Mouse	#554929	1:100	BD Bioscience
Anti-CD32-PE	Mouse	#552189	1:100	BD Bioscience
Anti-CD206	Rabbit	#ab64693	1:100	Abcam
Anti-rabbit-PE	Donkey	#12-4739-81	1:100	BD Bioscience

**Table 2:** Quantification for Western blot.

Antibody	Cell Lysate	Large-EVs	Small-EVs
Anti-TSG 101	1	0.69	3.20
Anti-Flotillin-1	1	9.42	41.78
Anti-Alix	1	3.30	35.70
Anti-HSC70	1	0.82	1.08

Protein levels for cell lysate (CL) were normalized to 1.0 and data were relative to CL.

**Table 3:** Statistics for Figure 4.

Target	ME [LPS]	ME [S-EV Tx]	Interaction
**TNF-α**	F(3,16) = 124.5; *p* < 0.0001	F(1,16) = 122.5; *p* < 0.0001	F(3,16) = 42.4; *p* < 0.0001
**IL-6**	F(3,16) = 988.4; *p* < 0.0001	F(1,16) = 406.5; *p* < 0.0001	F(3,16) = 239.3; *p* < 0.0001
**MCP-1**	F(3,16) = 288.4; *p* < 0.0001	F(1,16) = 163.9; *p* < 0.0001	F(3,16) = 82.9; *p* < 0.0001
**iNOS**	F(3,13) = 1133.0; *p* < 0.0001	F(1,13) = 643.8; *p* < 0.0001	F(3,13) = 229.4; *p* < 0.0001
**IL-10**	F(3,14) = 235.3; *p* < 0.0001	F(1,14) = 1239.2; *p* < 0.0001	F(3,14) = 53.75; *p* < 0.0001

Main effect (ME); concentration of LPS [LPS]; S-EV treatment [S-EV Tx].

## Data Availability

Not applicable.

## Supplementary Materials

The following are available online at https://www.mdpi.com/article/10.3390/biology10100948/s1, Figure S1: Microglia purity, Figure S2: Effect of neuronal L-EVs on microglia activity, Figure S3: To determine if L-EVs had the similar effects to suppress LPS-induced microglia activation as S-EVs, primary microglia were pre-incubated with S-EVs or L-EVs for 24 h and then treated with LPS (1 ng/ml) for 8 h, Table S1: Primary antibodies.
