# Supplementary material for: Neuron-Derived Extracellular Vesicles Modulate Microglia Activation and Function
Source: Biology (Basel). 2021 Sep 22;10(10):948. doi: 10.3390/biology10100948 (PMC8533634; doi:10.3390/biology10100948)
Supplement: Supplementary file 1 [file biology-10-00948-s001.zip › biology-1350679-supplementary.pdf]

Supplementary information, Figure S1.

To assess microglia purity, isolated cells were plated on 24-well plate at a density of 1 × 10^5 cells per ml for 24 h at 37°C and 5% CO_2 in a humidified incubator. Cells were incubated with anti-Iba-1 (microglia-specific marker, green, **A**) or anti-GFAP (astrocyte-specific marker, red, **B**), and nuclei were labeled with Hoechst 33258 (blue, **A** and **B**) as described in supplementary method, below. **C)** No NG2+ or MBP+ (Oligodendrocyte-specific markers) cells were observed in the microglial cell culture. **D)** NG2/MBP antibody efficacy was additionally verified in primary oligodendrocyte culture (see method below) where oligodendrocytes or oligodendrocyte precursors were immunoreactive for anti-NG2 (Oligodendrocyte precursor marker, red) and anti-MBP (Oligodendrocyte marker, green, **D**). For cell counting, cells were visualized under a Olympus IX81 fluorescent microscope with 20x objective.

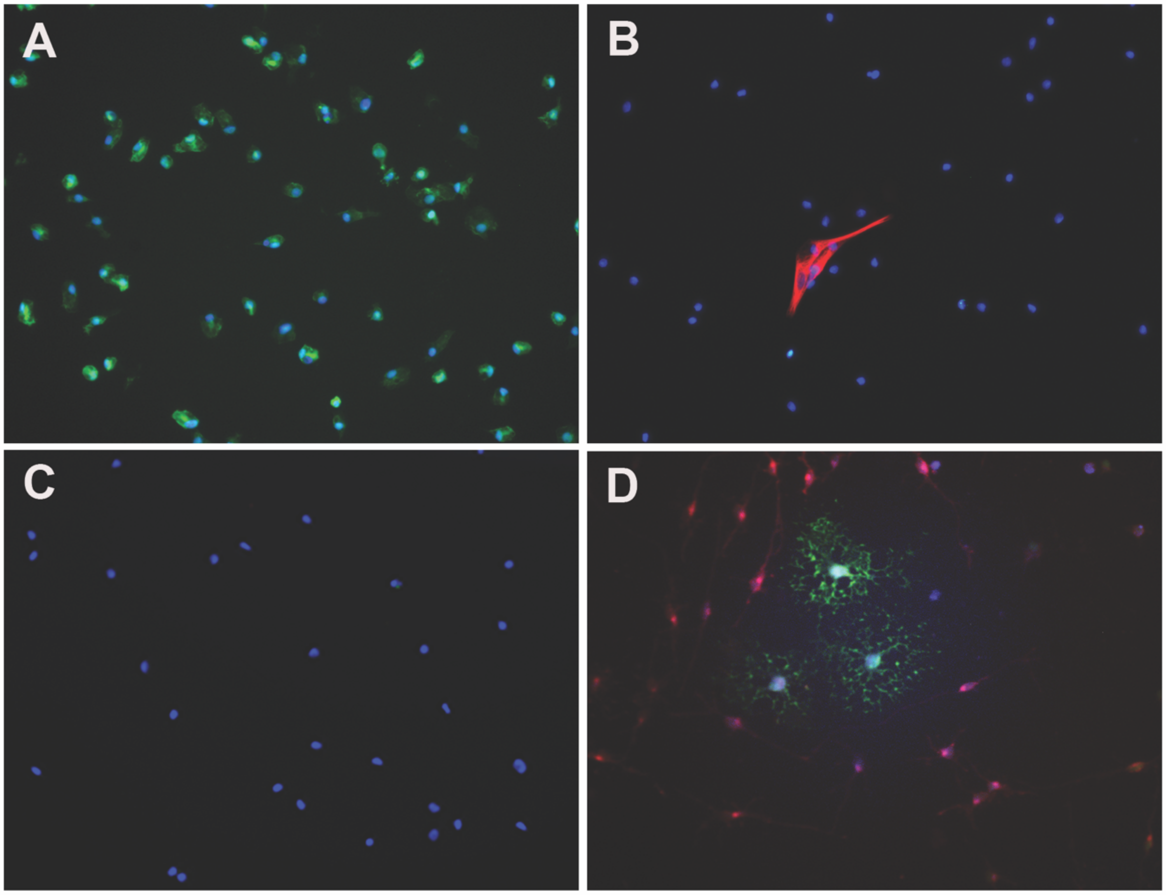

Supplementary information, Figure S2.

To determine the effect of neuronal L-EVs on microglia activity, primary microglia cultures were treated with S-EVs or L-EVs purified from rat cortical neuron culture. Twenty-four hours later, microglia were scraped from culture dishes and stained with microglia surface antigens, CD11b and CD32. Fixed cells were then analyzed by flow cytometry. Results showed that S-EVs reduced mean fluorescence intensity (MFI) of CD11b and CD32 and the expression frequency of CD32, while L-EVs had little effect on the MFI of CD11b or CD32, or the expression frequency of CD32.

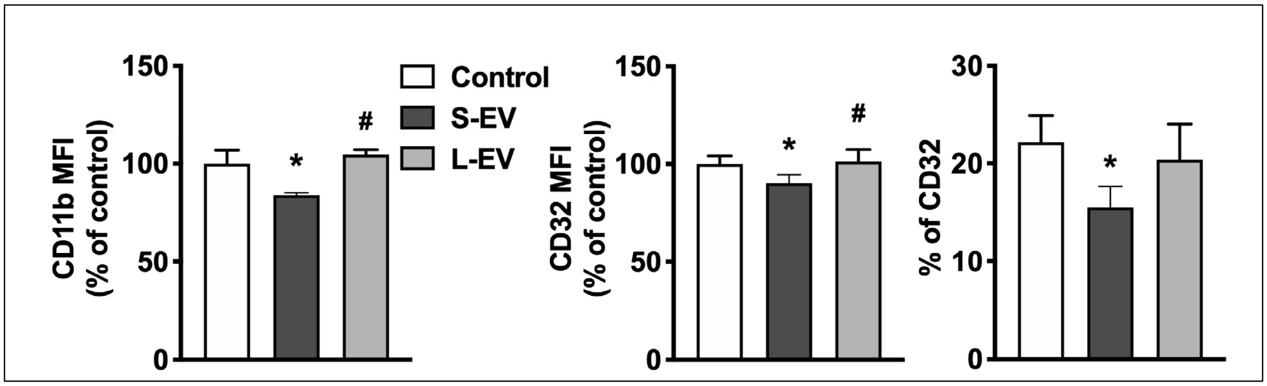

Supplementary information, Figure S3.

To determine if L-EVs had the similar effects to suppress LPS-induced microglia activation as S-EVs, primary microglia were pre-incubated with S-EVs or L-EVs for 24 h and then treated with LPS (1 ng/ml) for 8 h. Total RNA were extracted, and the levels of mRNA encoding pro-inflammatory cytokine/chemokines (TNF-α and MCP-1), iNOS, and anti-inflammatory cytokine (IL-10) were quantified by real-time RT-PCR. Results showed that L-EVs pre-treatment did not inhibit LPS-induced TNF-α, MCP-1, or iNOS expression, and did not promote IL-10 expression in microglia. Data are mean fold of control $\pm$ SD and analyzed by ANOVA. *p<0.05 versus control. # p<0.05 versus S-EV.

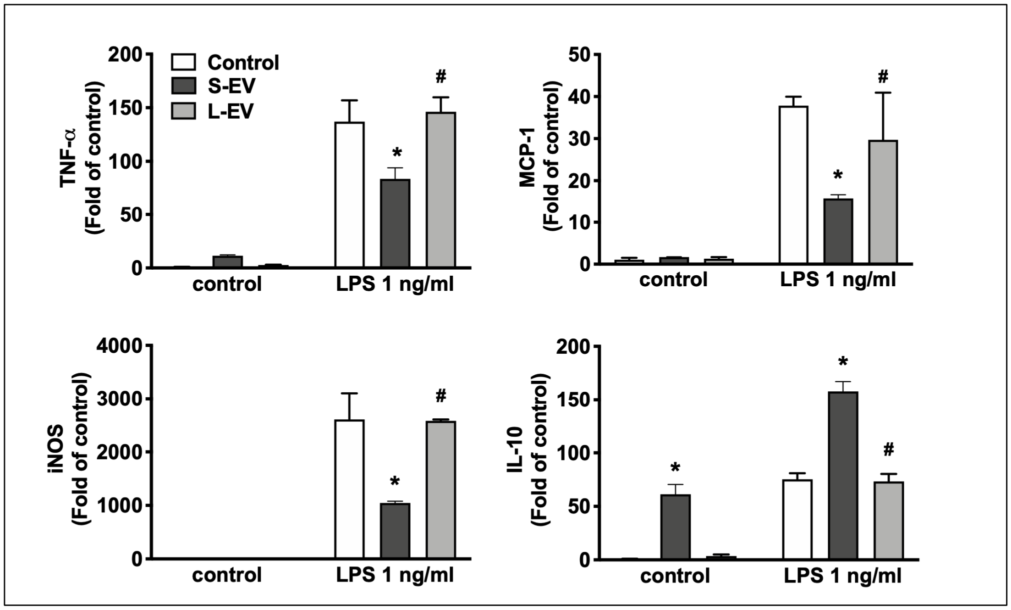

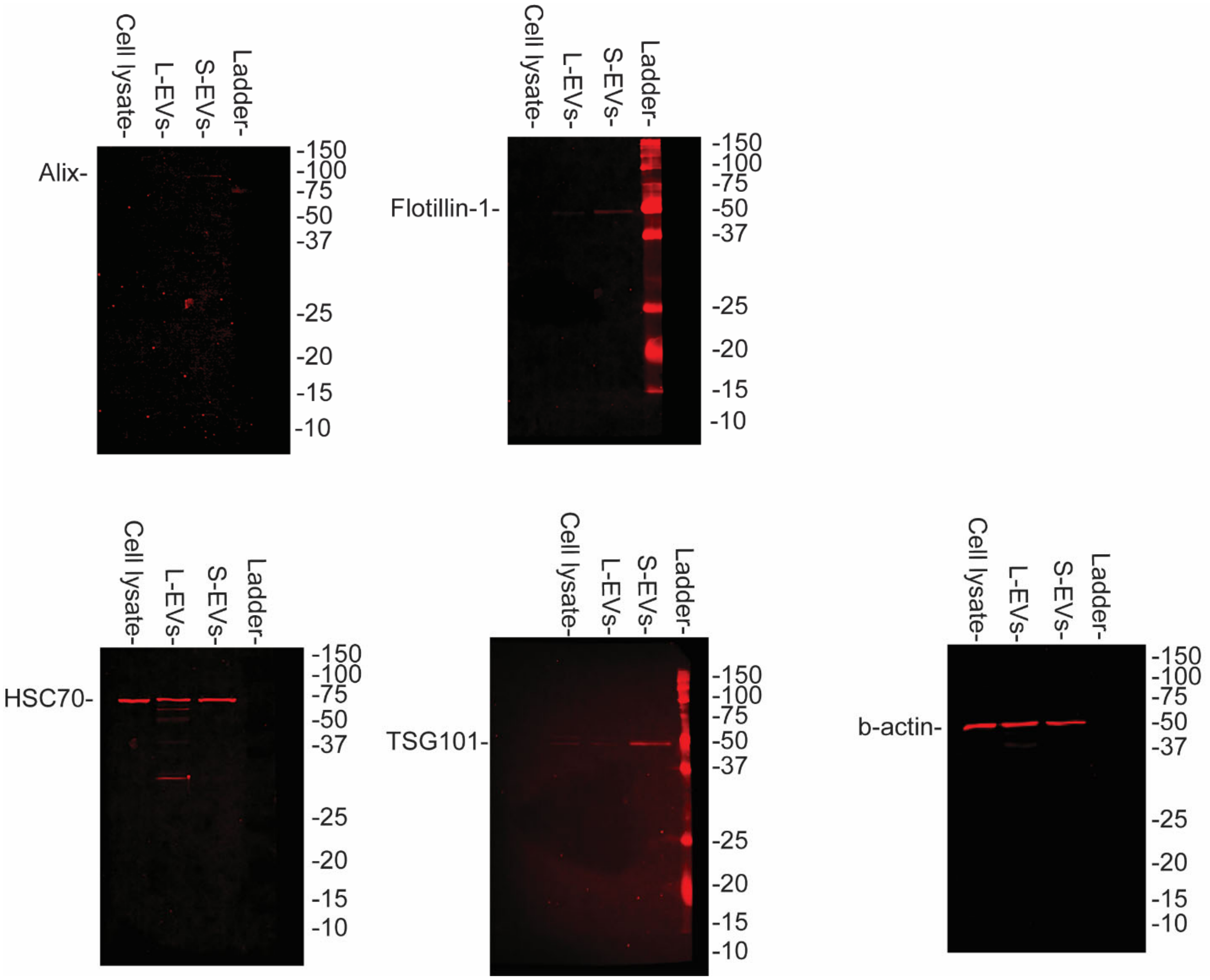

Western Blot Figure

Supplementary Method

Immunocytochemistry of cultured microglia and oligodendrocytes

Cells (microglia or oligodendrocytes) were rinsed with PBS and then fixed with 4% paraformaldehyde for 20 min at RT. The fixed cells were incubated overnight at 4˚C with antibodies for Iba-1, GFAP, NG2 or MBP (Supplemental Table 1) diluted in PBS containing 1 % BSA and 0.1% Triton X-100. Cells were then rinsed and incubated for 2 hr at RT with 1:400 anti-mouse, anti-rabbit or anti-goat IgG conjugated with Alexa 488 or Alexa 564. Nonspecific interactions of secondary antibodies were verified by omitting the primary antibodies. Nuclei were labeled with Hoechst 33258 (1 ug/ml for 10 min at RT). At least three independent microglial cultures were examined; five microscopic fields were counted per well and two wells per culture.

Supplemental Table S1: Primary antibodies

Antibody	Host species	Catalogue No.	Dilution	Resource
Iba-1	Goat	Ab5076	1:200	Abcam
GFAP	Rabbit	Z0334	1:1000	DAKO
NG2	Mouse	ZMS 1037	1:1000	Sigma
MBP	Rabbit	MAB386	1:1000	Sigma

Primary oligodendrocyte cultures

All procedures were in accordance with the Guide for the Care and Use of Laboratory Animals and were approved by the University of Kentucky Institutional Animal Care and Use Committee prior to the start of experiments. Rat pups were sacrificed on postnatal day 2 by rapid decapitation and the cortices were removed. The tissue was triturated in 0.1% trypsin (Sigma). Cells were resuspended and plated in poly-D-lysine-coated flasks for 8-10 d in DMEM containing 10% Fetal Bovine Serum (Thermo Fisher) and 1% penicillin/streptomycin. Microglia were reduced in cultures by gentle shaking at 230 rpm at 37˚C for 3 hr. The remaining oligodendrocyte progenitors presenting on the top of the confluent monolayer of astrocytes were detached by shaking overnight at 260 rpm as described[1]. The enriched oligodendrocyte progenitor cell suspension was plated onto poly-D-lysine-coated 24-well plate in serum-free defined medium containing 5 μg/ml PDGF-AA and bFGF (RnD Systems) for 2 days as described[1]. Cells were switched to serum-free defined medium without growth factors for additional 5 day to promote the differentiation of oligodendrocyte progenitors to MBP-positive oligodendrocytes.

Reference:

[1] Molina-Holgado E, Vela JM, Arevalo-Martin A, Almazan G, Molina-Holgado F, Borrell J, Guaza C: Cannabinoids promote oligodendrocyte progenitor survival: involvement of cannabinoid receptors and phosphatidylinositol-3 kinase/Akt signaling. J Neurosci 2002, 22:9742-53.
